# Temporal Variation of Wood Density and Carbon in Two Elevational Sites of *Pinus cooperi* in Relation to Climate Response in Northern Mexico

**DOI:** 10.1371/journal.pone.0156782

**Published:** 2016-06-07

**Authors:** Marín Pompa-García, Alejandro Venegas-González

**Affiliations:** 1 Facultad de Ciencias Forestales, Universidad Juárez del Estado de Durango, Río Papaloapan y Blvd. Durango s/n, col. Valle del Sur, 34120, Durango, Durango, México; 2 Department of Forest Sciences, Wood Anatomy and Tree-Ring Laboratory, University of São Paulo, Piracicaba, 13418900, São Paulo, Brazil; University of Vigo, SPAIN

## Abstract

Forest ecosystems play an important role in the global carbon cycle. Therefore, understanding the dynamics of carbon uptake in forest ecosystems is much needed. *Pinus cooperi* is a widely distributed species in the Sierra Madre Occidental in northern Mexico and future climatic variations could impact these ecosystems. Here, we analyze the variations of trunk carbon in two populations of *P*. *cooperi* situated at different elevational gradients, combining dendrochronological techniques and allometry. Carbon sequestration (50% biomass) was estimated from a specific allometric equation for this species based on: (i) variation of intra-annual wood density and (ii) diameter reconstruction. The results show that the population at a higher elevation had greater wood density, basal area, and hence, carbon accumulation. This finding can be explained by an ecological response of trees to adverse weather conditions, which would cause a change in the cellular structure affecting the within-ring wood density profile. The influence of variations in climate on the maximum density of chronologies showed a positive correlation with precipitation and the Multivariate El Niño Southern Oscillation Index during the winter season, and a negative correlation with maximum temperature during the spring season. Monitoring previous conditions to growth is crucial due to the increased vulnerability to extreme climatic variations on higher elevational sites. We concluded that temporal variability of wood density contributes to a better understanding of environmental historical changes and forest carbon dynamics in Northern Mexico, representing a significant improvement over previous studies on carbon sequestration. Assuming a uniform density according to tree age is incorrect, so this method can be used for environmental mitigation strategies, such as for managing *P*. *cooperi*, a dominant species of great ecological amplitude and widely used in forest industries.

## Introduction

The forest growth is one of the most important processes that determine the carbon balance of terrestrial ecosystems [1]. In this sense, forest dynamics are strongly associated with ecological processes that occur over a forest′s lifetime, such as climate [2], management regimes and ecological niches [3], among others, are factors that determine their magnitude. Recently, several studies have been conducted globally to understand carbon fluxes on temporal and spatial scales [4–8]. Dendrochronology facilitates understanding the dynamics of tree growth and its relationship with associated influences [9]. In addition to the spatial perspective they provide, elevation gradients are ideal for such studies [10–12].

Consequently, combining dendrochronological techniques and allometric equations can enhance understanding of carbon fluxes and their climatic sensitivity [1,2,13]. Traditionally, biometric studies and strategies for limiting damage to forest ecosystems (e.g., reducing emissions from deforestation and forest degradation) use density and diameter to estimate biomass content [14,15]. Thus, it is assumed that the density is constant, even though this parameter varies temporally, depending on tree age [16–19]. This results in differential rates of carbon, with variations occurring among species and ecosystems. Thus, we can test the hypothesis that carbon sequestration varies over time and in different ecosystems [20,21]. This should help overcome theoretical limitations in understanding carbon fluxes and gaining accurate knowledge of forest productivity.

Mexican forests have the largest number of native pines worldwide [22]. *Pinus cooperi* is a dominant species, of great ecological amplitude, and its dendrochronological potential has been verified [23]. However, the state-of-the-art findings from the literature appear incomplete for Mexican forests. Studies using a combination of densitometry and allometric techniques to understand the temporal and spatial dynamics of carbon have not been reported for those forests. This study attempts to fill in this gap in the literature. We analyzed the variations of carbon in two populations of *Pinus cooperi* situated on different elevation gradients, using intra-annual wood density. The relationship between growth rings and climatic factors was also analyzed, based on maximum latewood density.

## Methodology

### Study area and data

The study area corresponds to two *Pinus cooperi* stands in the Sierra Madre Occidental of northern Mexico (Table 1). These stands are at sites High (H) and Higher (HE), which are in altitude gradients above 2600 msnm, where tree growth was strongly associated with climate in previous research [24]. Respect to ethics statement, this study was conducted on public land known as communally held land, which is technically managed by Forest Engineer Jose Santiago Sanchez Huerta. Mr. Sanchez is accredited by the Mexican Federal Government agency SEMARNAT (i.e. Secretariat of Environment and Natural Resources). This species is not endangered or threatened.

**Table 1:** Descriptive data of sample plots of *Pinus cooperi*.

Site	Long W (°)	Lat N (°)	Elevation (masl)	Trees	Mean Dbh (cm)
**Higher-HE**	105.534944	23.747306	2905	15	47.2
**High-H**	105.48828	23.72436	2680	16	50.1

Trees were growing under marginal environmental conditions characterized by thin and rocky soils, moderate slopes (5–10%), and southeastern exposure. The area has a temperate sub-humid climate, with a wet and cool summer (June–September) caused by monsoons and characteristic dry seasons in spring (March–May) and winter (October–February) [23].

Given its transitional location between Holartic and Neotropical zones and the complexity of its physiography and climate, the study area has a great variety of ecosystems, which are dominated by pine and/or oak forests [25]. A cool and wet climate prevails in most of the forest stands, with an average annual rainfall of 950 mm. Most precipitation occurs from June through September. Mean January and July temperatures are 7°C and 15.5°C, respectively. Altitude ranges from 2600 to 3100 m. Natural vegetation consists of mixed conifer forests dominated by *P*. *cooperi* and other species of *Pinus (P*. *durangensis*, *P*. *leiophylla*, *P*. *strobiformis and/or P*. *pseudostrobus*), which are often found together with *Quercus* and *Arbutus* species. Important understory shrubs include *Garrya wrightii*, *Ribes ceriferum*, and *Vaccinum geminiflorum*. Ground cover consists of various species of grasses, herbs and ferns.

### Annual wood density measurement

At each of the two sites, at least two increment cores were collected from a height of 1.3 m by a non-destructive method. Tree ring samples were collected from 15 to 16 trees per site (Table 1). Wood cores were cut in the transverse direction, maintaining a thickness of 1.7 ± 0.02 mm, and were kept in a conditioning room at 20°C and 50% relative humidity until reaching a stable moisture content of 12% [26]. To determine annual ring widths and wood densities, wood samples were scanned from bark to pith using an X-ray densitometry QTRS-01X Tree Ring Scanner (Quintek Measurement Systems, Knoxville, TN, USA) at 0.04-mm intervals. The demarcation zone among tree rings was automatically set up by the device and checked manually for every tree-ring scanned.

We determined the following for each year: tree-ring width (RW), earlywood width, and latewood width, mean density (MD), minimum and maximum density (MXD). However, only RW, MD and MXD data were used in this study. RW was used to quality-check the cross-dated tree-ring sequences using the software program COFECHA (named after the invented Spanish word *cofecha*, meaning "co-date" or "cross-date ") [27]. MD was used to estimate aboveground woody biomass increment [13] and MXD used to analyze tree response to local and global climatic variability [28].

### Chronology development

Cores were dated visually during the X-ray densitometry, and by observing the difference between earlywood and latewood; then densitometry data for each ring at resolution 0.04 mm were recorded. To remove non-climatic trends of tree growth from the TRW and MXD measurement series, we used the program ARSTAN (AutoRegressive STANdardization) [29]. Each series was detrended using a cubic spline with 50% frequency-response with a cutoff equal to 2/3 of series length, while preserving high-frequency (yearly to sub-decadal) climatic information and removing low-frequency trends in the series. The regional tree-ring chronology (HE + H) was calculated by averaging the MXD series with a bi-weight robust mean estimation in ARSTAN. Detrending was followed by transforming tree-ring widths to dimensionless growth indexes, by dividing observed ring-width values by fitted ones. Standard and residual indices were calculated.

The quality of the chronology was tested via the following statistics: mean sensitivity (MS), which measures interannual variability in tree rings; expressed population signal (EPS), which measures the strength of the common signal in a chronology over time and verifies the hypothetically perfect chronology; average correlation between all series (RBAR), which measures the common variance between individual series in a chronology; first-order autocorrelation (AR1), which is a measure of the association between tree-ring growth in two consecutive years [30]. RBAR and EPS were run using 20-year moving windows with 10-year overlaps. EPS values > 0.85 are generally accepted as a common signal between trees [31].

### Wood density-climate relationship

The influence of climate on MXD of *P*. *cooperi* was assessed by Pearson's correlation coefficient analysis. To evaluate the relationship between MXD and seasonal climatic factors (summer months: JJA; fall: SON; winter: DJF; spring: MAM), we investigated that coefficient with the program DENDROCLIM2002 [32]. This software applies statistical significance of Person’s correlation coefficients by calculating 95% limits based on 1000 bootstrapped resamples of the data. Correlation coefficients were calculated for each site using residual chronologies, because values of first-order autocorrelation of the two populations were overly large. Thus, series were pre-whitened by autoregressive models to remove temporal autocorrelation [29]. Chronologies were compared with local climate variability, i.e., maximum temperature, minimum temperature and total monthly precipitation from 1946–2014, obtained from the meteorological station "El Salto" (Comisión Nacional del Agua). The chronologies were also compared with global climate variability, i.e., multivariate El Niño Southern Oscillation index (MEI), based on the six main observed variables over the tropical Pacific [33], sea level pressure (SLP), zonal (U) and meridional (V) components of the surface wind, sea surface temperature (SST), Air temperature (AT), and total cloudiness fraction of the sky (C). The MEI data were retrieved from NOAA for each of twelve sliding bimonthly seasons (http://www.esrl.noaa.gov/psd/enso/mei/table.html). In addition, to estimate the association between MXD and MEI, we built a correlation map between MXD regional chronology and five variables of MEI (SLP, U, V, SST and AT) for the tropical pacific region, using 2.5 × 2.5 grid cells from the National Centers for Environmental Prediction reanalysis global dataset [34]. SST, AT and SLP were analyzed at surface level, and U and V at 250-hPa geopotential height (U250 and V250).

### Aboveground woody biomass (carbon)

Tree-ring width annual values (radial growth) were used to reconstruct historical tree diameters and their basal area increment (BAI). Along with mean wood density (MD), these values were used to estimate biomass and, thus, carbon accumulation of *P*. *cooperi* (CA). We used the allometric equation for biomass estimation of northwestern Mexico forests [15], which was constructed to estimate carbon stocks for forests of northwestern Mexico:

$$AWB\;=\;0.0752*D^{2.4448}*2.0331^{p},$$

where *AWB* = aboveground woody biomass, *D* = diameter at breast height, and *p* = wood density. We considered a 50% carbon content of woody biomass [35].

We performed a statistical analysis using the Wilcoxon–Mann–Whitney (W) test, with *P* < 0.05 for significance, to evaluate differences of basal area increment, mean wood density and uptake carbon between the sites. We used a non-parametric analysis because the values do not entail the basic assumptions of a normal distribution, according to the Shapiro–Wilks test [36].

## Results

### Chronology characteristics

**Fig 1:**
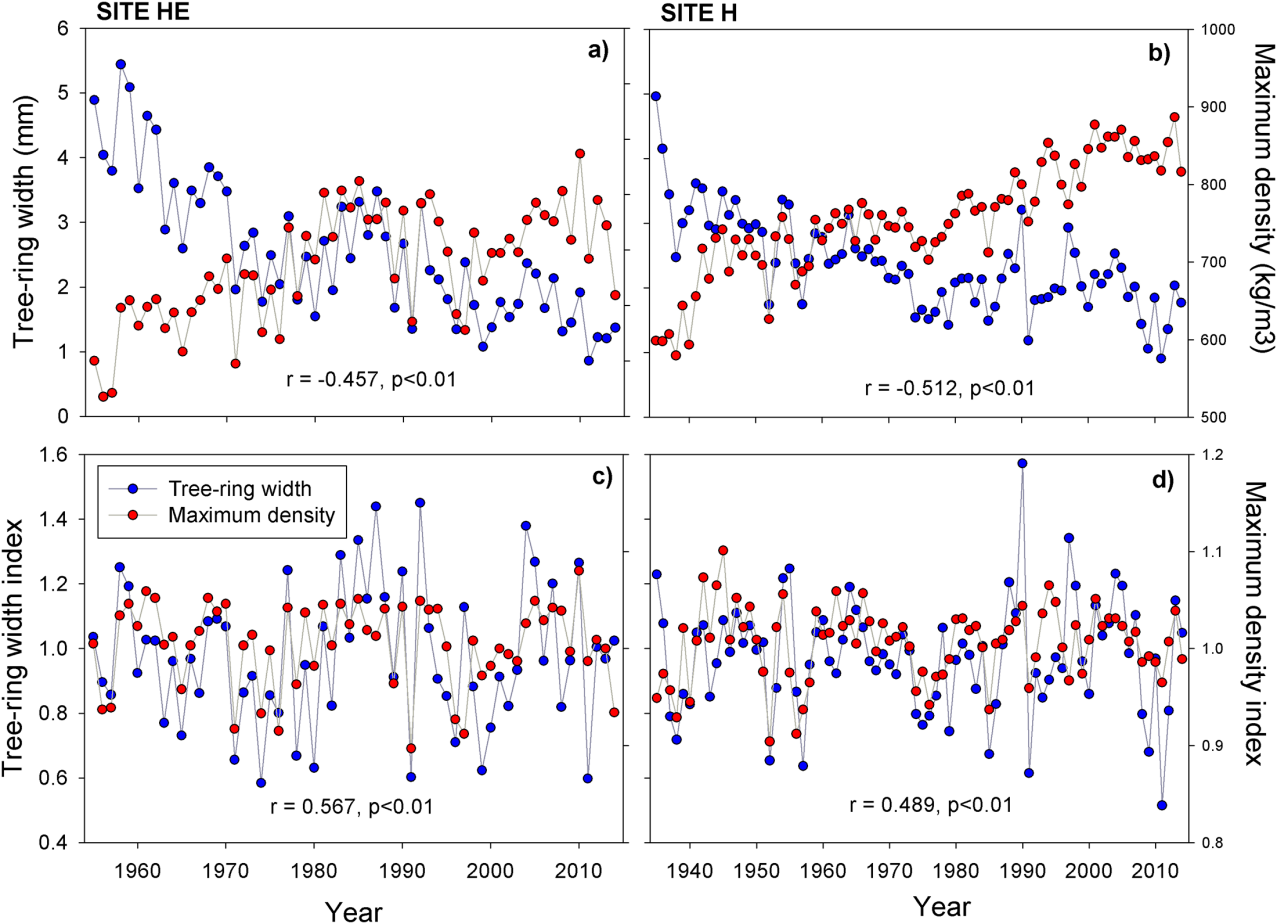
Time series of tree-ring width and maximum latewood density. (a), (b) Chronologies of tree-ring parameters without standardization. (c), (d) Chronologies of tree-ring parameters detrended of *P*. *cooperi* trees at sites H and HE for the period 1935–2014 and 1955–2014, respectively.

At both sites, there was a negative correlation between mean series of tree-ring width and MXD (*p* < 0.01). However, for detrended values, the correlation was positive (*p* < 0.01), demonstrating the importance of detrending the series (Fig 1). The length of two chronologies (RW and MXD) was 60 years for HE and 80 years for H, considering at least five trees (Table 2; Fig 1). Mean tree-ring width and wood density rates were 2.60 ± 1.43 mm years^−1^ and 0.77 ± 0.12 g cm^−3^ years^−1^, respectively, with the HE population having the larger values of the two variables. Mean sensitivity in RW was > 0.30, showing that trees react to the environment through their annual growth variability [27]. Both RW and MXD had large 1st-order autocorrelation values, demonstrating the importance of choosing the residual chronology in this species. In general, the mean RBAR of both variables is small in the two populations. MXD showed lesser sample quality than RW, demonstrated by EPS values across the chronology. Those values were ≥0.85 over the entire period for both populations, showing that sampling replication was adequate [31] (Table 2). There is substantial variance explained by the first principal component (PC1) of both variables in the regional chronology, according to the regional mean (RW PC1 = 60.8; MXD PC1 = 42.1).

**Table 2:** Statistical characteristics of chronologies of *P*. *cooperi*.

Site	Variable	TS	Mean ± SD	SI	MS	RBar	AC1	EPS	PC1
**HE**	RW	1955–2014	2.98 ± 1.48	0.56	0.39	0.46	0.55	0.93	78.7
	MXD		0.78 ± 0.08	0.36	0.10	0.25	0.42	0.84	44.3
**H**	RW	1935–2014	2.51 ± 1.21	0.45	0.35	0.22	0.59	0.85	43.0
	MXD		0.75 ± 0.11	0.18	0.10	0.10	0.60	0.65	39.8
**Regional**	RW	1935–2014	2.60 ± 1.43	0.43	0.37	0.23	0.57	0.89	60.8
	MXD		0.77 ± 0.12	0.26	0.10	0.12	0.52	0.82	42.1

H: site high; HE: site higher; RW: tree-ring width; MXD: maximum latewood density; TS: time span considering at least five trees; Mean± SD: mean ring width ± standard deviation (mm and gcm^-3^); SI: series intercorrelation; MS: mean sensitivity; AC: first-order autocorrelation; RBAR: mean inter-series correlation, EPS: Expressed population signal, PC1: variance explained by the first principal component.

### Climatic influence on maximum latewood density (MXD)

**Fig 2:**
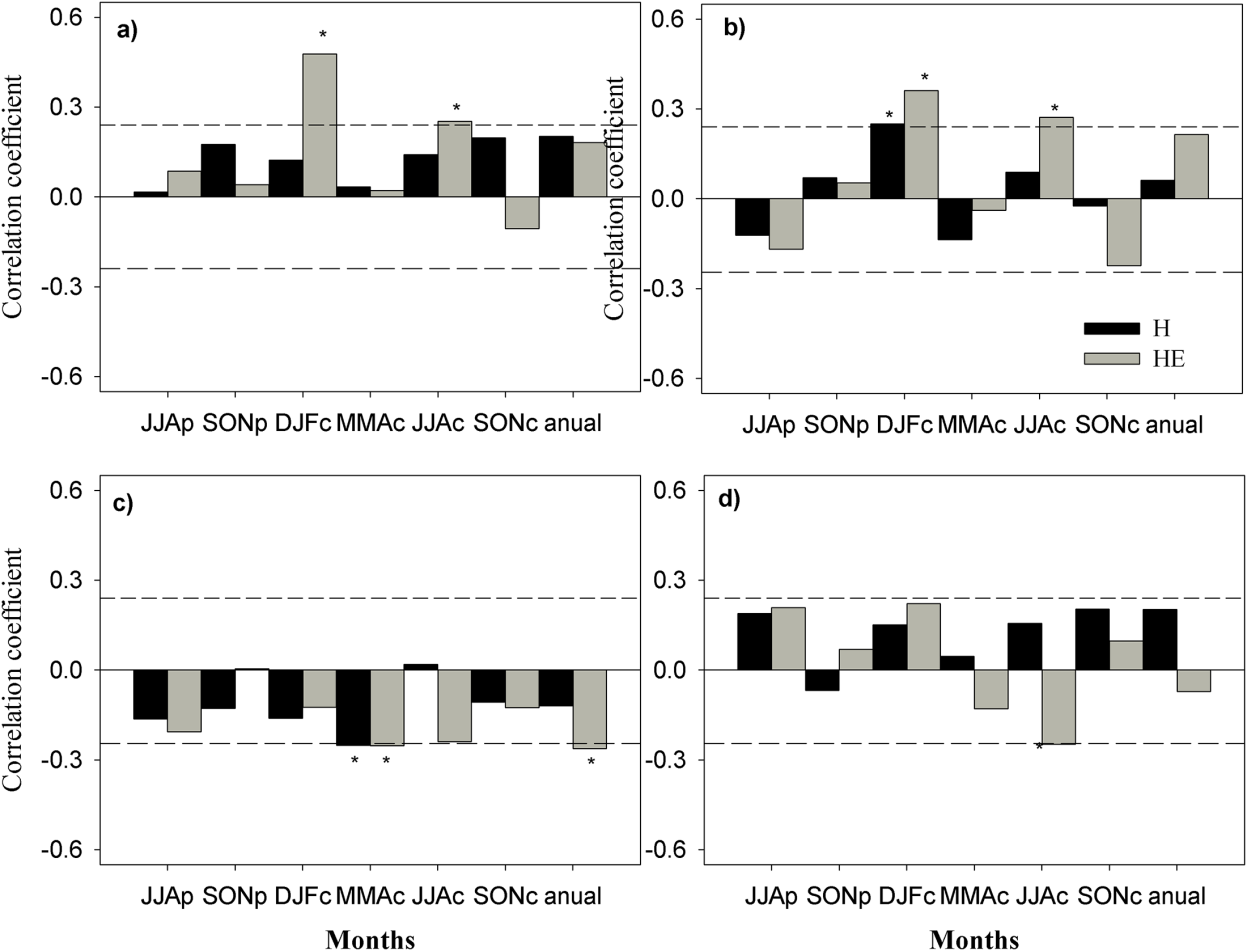
Relationships between maximum latewood density (MXD) chronologies and climatic variables. (a) Accumulated precipitation (P). (b) Mean multivariate ENSO index MEI. (c) Temperature maximum (Tmax). (d) Temperature minimum (Tmin). Significant correlation coefficients (*p* < 0.05) are marked with asterisks. Letters indicate previous (p) and current (c) year. JJA = June to July, SON = September to November, DJF = December to February, and MAM = May to May.

The climate-MXD relationship is shown in Fig 2, for the period 1946–2010 for site H and 1955–2010 for site HE. In general, site HE was more influenced by the climate than site H. In terms of precipitation, both sites had a strong positive correlation with cold months before the start of the growing season; this correlation was significant in November, December and January at HE (N: *r* = 0.35, D: *r* = 0.32, J: *r* = 0.32; *p*<0.05) and December and February at H (D: *r* = 0.20, F: *r* = 0.22; *p*<0.05). However, analysis by season indicated that only site H showed a strong correlation with the cold season (DJF: *r* = 0.48; *p*<0.01). Maximum temperature showed negative correlation with all months at both sites, and was significant in spring (MAM: *r* = −0.25; *p*<0.05). Minimum temperature did not have a noticeable influence on MXD, except in summer at site HE, with a negative correlation (JJA: *r* = −0.25; *p*<0.05).

**Fig 3:**
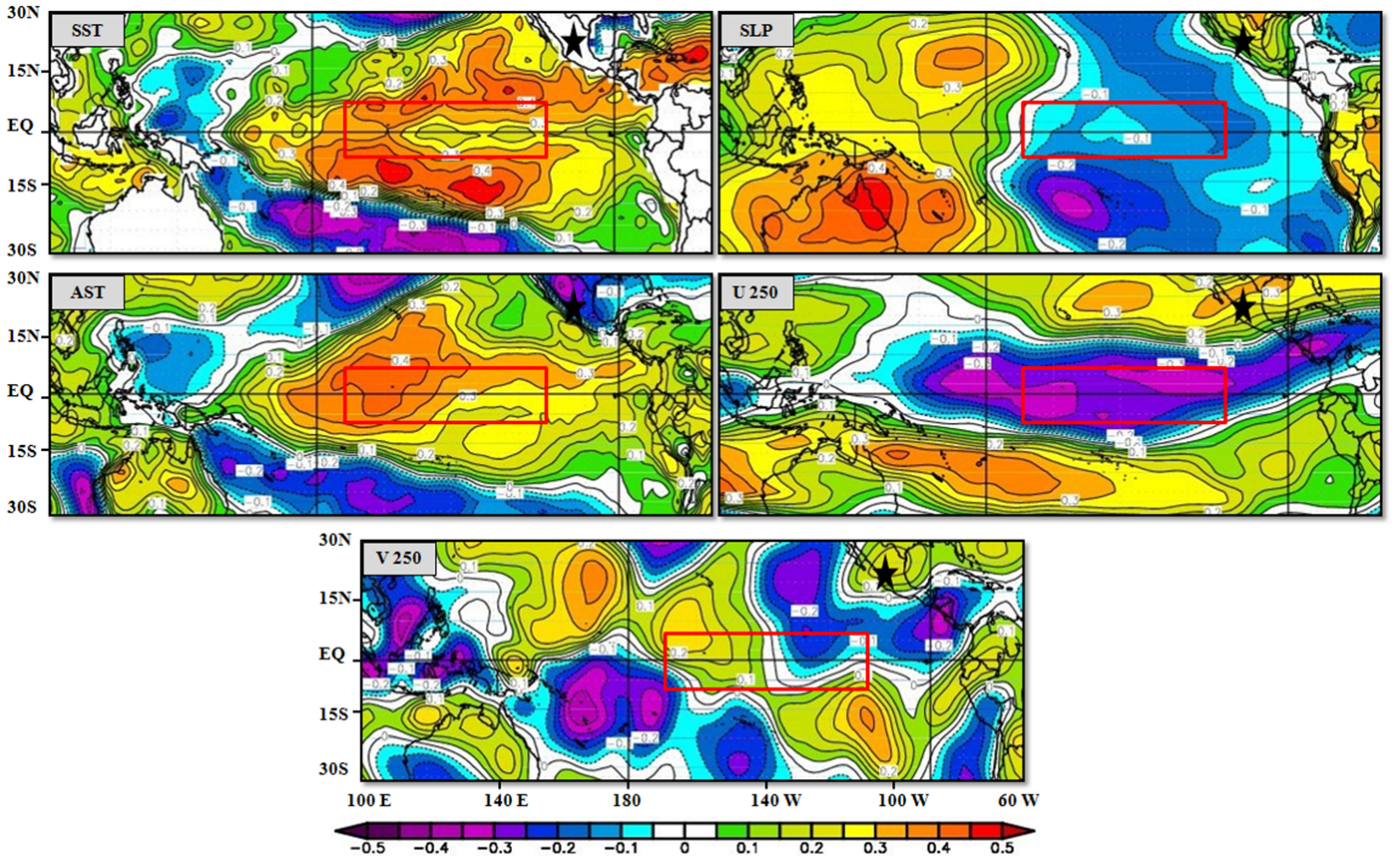
Spatial correlation between MXD regional chronology and MEI parameters. Spatial correlation between fields of MXD regional chronology and 2.5° × 2.5° gridded monthly average (December–February) of sea surface temperature (SST), sea level pressure (SLP), surface air temperature (SAT), 250 hPa zonal wind (U250), and 250 hPa meridional wind (V250), for the period 1948–2011. Correlation values are shown by color scale. Data were obtained from the National Oceanic and Atmospheric Administration website (www.esrl.noaa.gov/psd/data/correlation). Study area is indicated by a black star. 3.4 Niño region is represented by a red rectangle. Significant correlation at 90%; confidence level is r = 0.20.

In examining the influence of global climate factors on MXD using ENSO patterns, we observed an association between the MEI and MXD at both sites in the cold season, which would influence rainfall (H: *r* = 0.25, *p*<0.05; HE: *r* = 0.36, *p*<0.01). Spatial correlation showed a positive and significant association (p < 0.1) between MXD regional chronology and SST, SAT and V250, and a negative association with sea level pressure (SLP) and U250 in the N3.4 region (which encompasses parts of regions 3 and 4, and lies between 120W°–170°W and 5°N–5°S), over the period 1948–2014 (Fig 3).

### Estimation of uptake of annual and cumulative carbon

**Fig 4:**
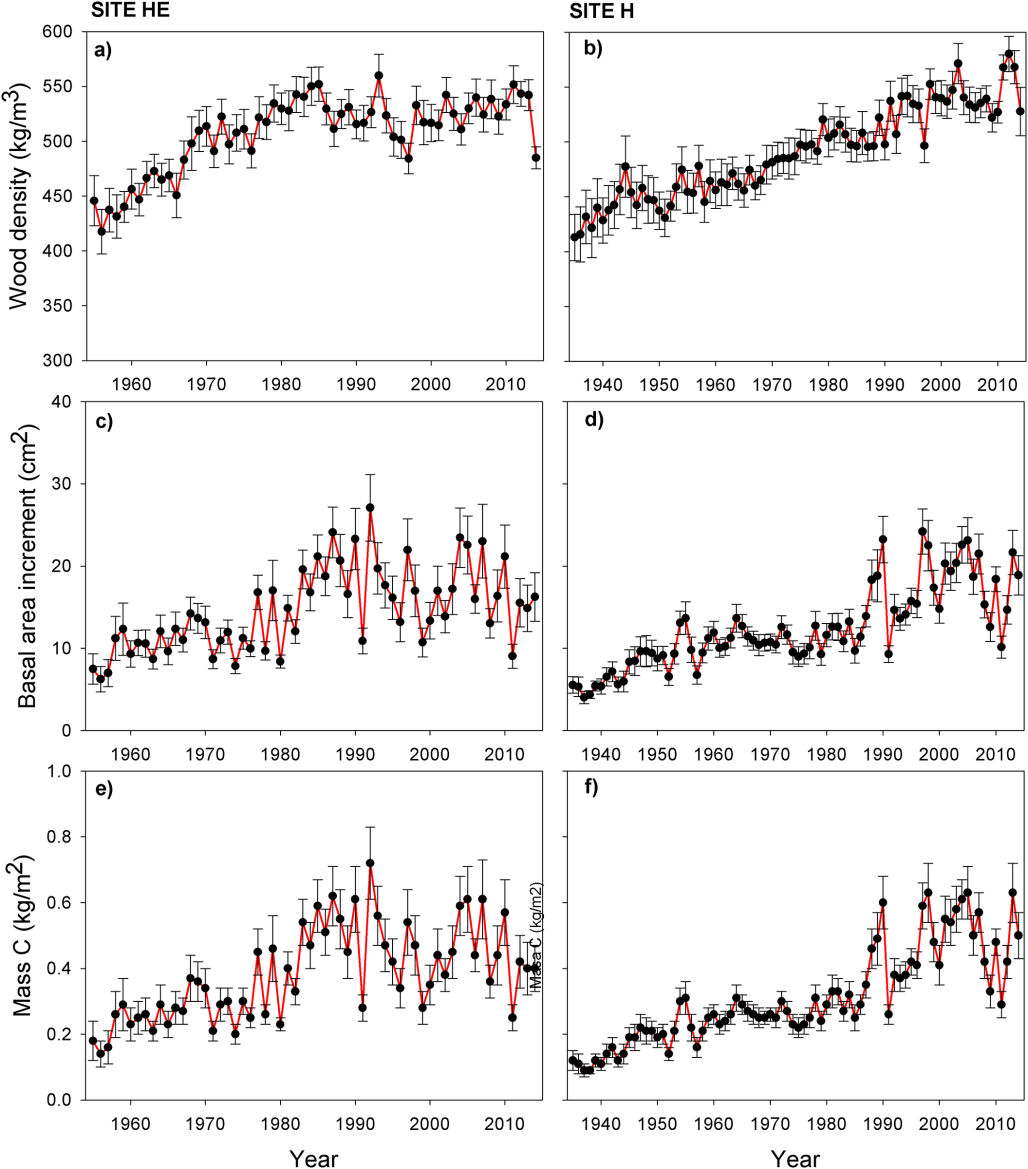
Inter-annual variability of variables related to biomass accumulation (mean ± SD). (a), (b) Wood density. (c), (d) Basal area increment. (e), (f) Annual carbon uptake of *P*. *cooperi* trees at sites H and HE for the period 1935–2014 and 1955–2014, respectively.

**Fig 5:**
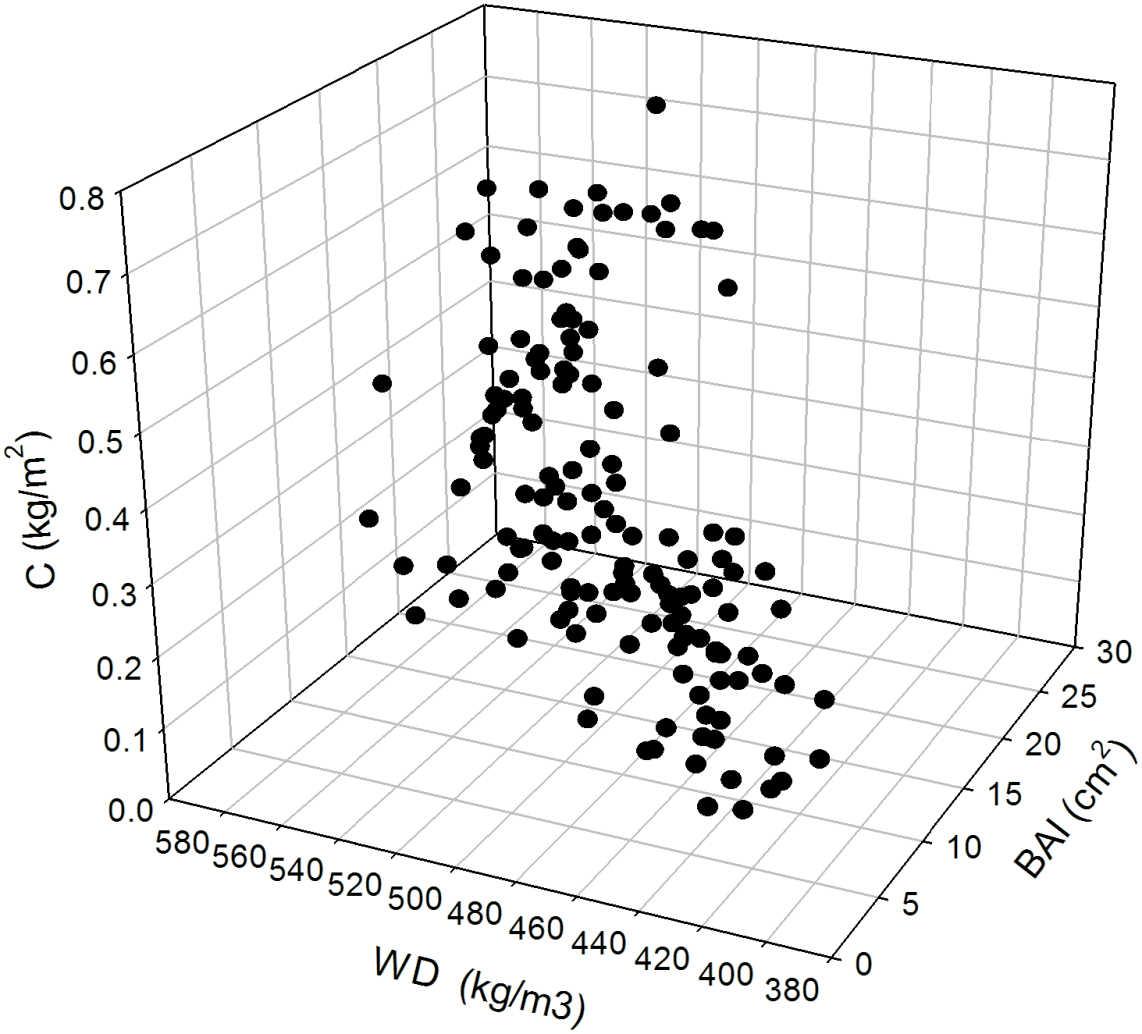
3D scatter plot of variables related to biomass accumulation. Mean values of annual carbon uptake (C), annual mean basal area increment (BAI) and annual mean wood density (WD) of of *P*. *cooperi* trees at sites H and HE.

Wood density tended to increase with age, with larger values for tree rings near bark (Fig 4a and 4b). Basal area increment was ~10 cm^2^ in the early years to mid-1970s at HE and in the mid-1980s at H; it reached 20 cm^2^ year^−1^ in both populations, with peaks close to 30 cm^2^ year^−1^ in 1992 at HE and 1997 at H (Fig 4c and 4d). The annual carbon uptake curve was similar to the basal curve, verifying the positive relationship between the two variables (Fig 4e and 4f). In this sense, larger values of annual carbon uptake are directly associated with larger values of annual basal area increment and annual mean wood density (Fig 5).

**Fig 6:**
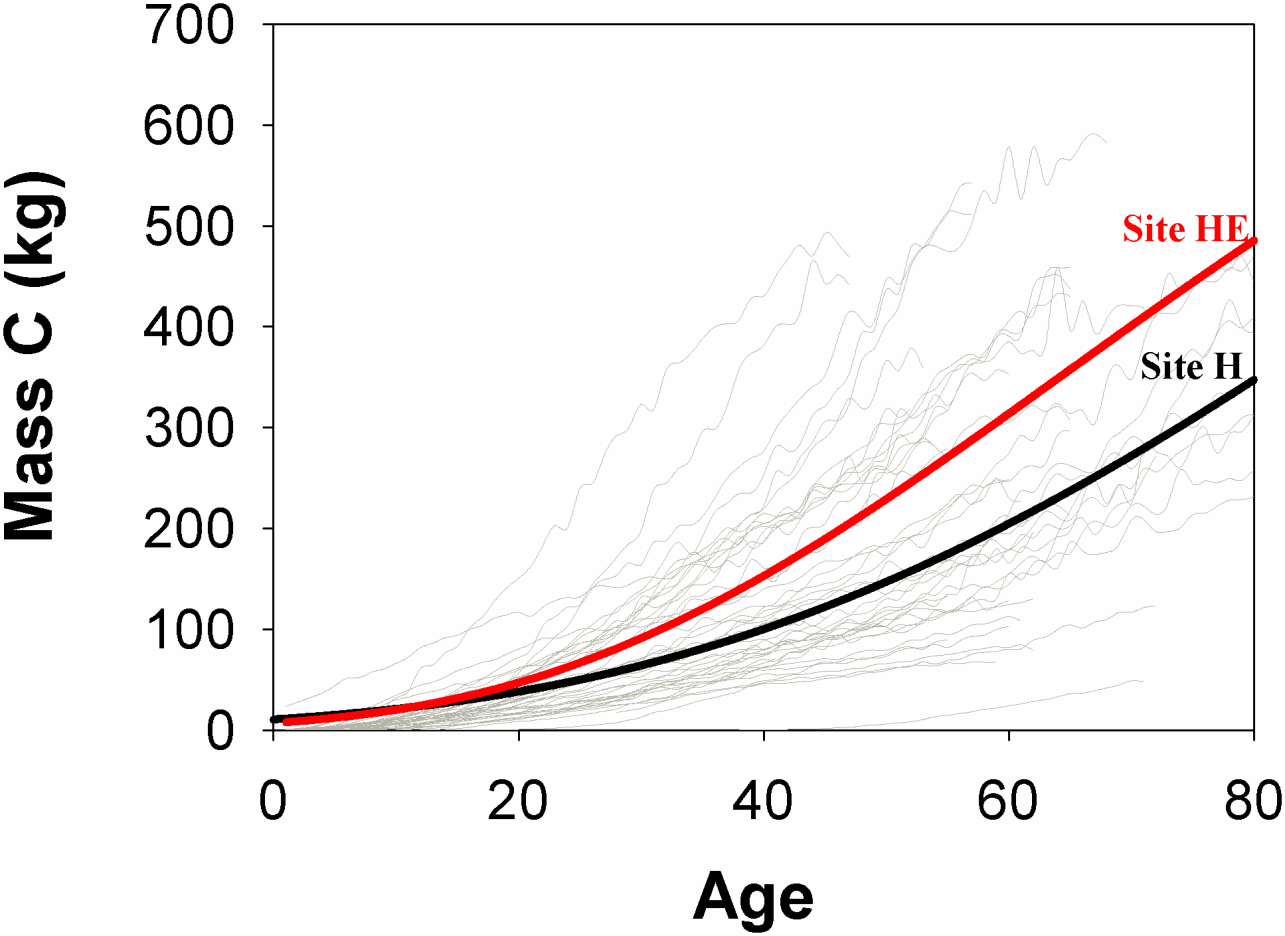
Cumulative carbon of aboveground woody biomass of *P*. *cooperi* trees. Gray lines represent cumulative carbon of cores. Black and red lines indicate cumulative carbon at H and HE sites, respectively, which are represented by sigmoid regression model (both R^2^ = 0.99).

*P*. *cooperi* trees at site HE showed greater cumulative carbon than at H during the analysis period; these were ~300 and 200 kg at 60 years, respectively, with a tendency to continue increasing (Fig 6). In addition, site HE had a significant difference in annual carbon uptake (Table 3). Although wood annual density and basal area increment were not statistically significant, both variables tended to be greater at HE than H.

**Table 3:** Mean ± SD of annual wood density (WD kg m^−3^), annual basal area increment (BAI cm^2^) and annual carbon uptake (C kg m^−2^) of *P*. *cooperi* trees.

Site^a^	WD	W	P^b^	BAI	W	P^b^	C	W	P^b^
**HE**	500.1 ± 39.8	5528	0.07	13.8 ± 5.2	5376	0.09	0.36 ± 0.15	5474	0.04
**H**	490.3 ± 41.2			12.4 ± 4.9			0.31 ± 0.15		

^a^H: site high; HE: site higher.

^b^Statistically significant difference is represented by *p* values < 0.05 determined by Wilcoxon–Mann–Whitney (W) test.

## Discussion and Conclusions

To our knowledge, the present study is the first to document about climate-trees relationship and estimate carbon using intra-annual wood density in *P*. *cooperi*. This study will support efforts to quantify carbon dynamics in temperate forests considering temporal variation of wood density. The incorporation of wood density improves aboveground biomass estimation (i.e. carbon uptake) [37]. We used values of annual density in allometric equation to quantify the magnitude and inter-annual variability in tree growth [13], since wood density varies temporally, depending on tree age [16–19] and diameter class [38]. Baker et al. [39] have shown that ignoring variations in wood density should result in poor prediction of the stand aboveground biomass and consequently in carbon calculation. Further, the association of wood density with climatic fluctuations herein significantly improves up on existing knowledge of trends in carbon sequestration within ecosystems [40].

### MXD Chronologies show a link to local climate

The results of the chronologies in this study are similar to those for the same species at neighboring sites [23,24]. This regional similarity suggests consistency with annual biomass increases (Fig 1). The divergent trends between tree-ring width and MXD shown in Fig 1a and 1b are consistent with their geometry and tree age, whereas the correlation between chronology trends and maximum density index (Fig 1c and 1d) suggests a potentially effective standardization method, as evidenced by the synchronized sequences between TRW and MXD. These trends are attributed to climatic factors [41]. Furthermore, most of the literature related to densitometry and climatic variables are based on MXD [28,42,43], since inclusion of this variable strengthens paleoclimatic studies [44]. Its positive correlation (Fig 1) indicates that in years with strong radial growth, there is an increase in density of latewood, i.e., an increase in tracheids of thicker cell walls, mainly in the tangential direction [45]. This result has also been reported in other studies [46].

This strong relationship is based on the duration of tracheid formation, which indicates thinner cell walls [47]. That is, MXD variation is strongly related to climate sensitivity, particularly seasonal variations of both temperature and precipitation, such as those occurring in the study area [48].

Gradual differentiation of precipitation and temperature produces differences in tree physiology [10]. This idea is confirmed by the results in Fig 2, which show that climate response varies with the specific gradient; that is, the higher-altitude site is more sensitive to the climate. Winter water conditions prior to growth restrict the radial development of *P*. *cooperi*, particularly at higher-elevation sites. It is well known that winter rain results in water recharge in *P*. *cooperi*, creating a positive balance compared with the dormancy period of the species. This recharge is useful for the start of the next growing season, when it improves the trees’ photosynthetic activity [49]. Studies have shown such an association between radial growth and winter precipitation in the Sierra Madre Occidental [25,50].

Regarding temperature, Lebourgeois et al. [51] indicated that optimal radial growth occurs at a mean temperature; however, here we observed that the two sites showed different responses to maximum temperature and minimum temperature. Temperature controls the number and size of cells in latewood density [41]. Specifically, high temperatures can contribute to latewood cell wall thickening, thus produce denser latewood [52], but it does not match our result (Fig 2c). Moreover, minimum temperature is beneficial for tree growth in the pre-growth season [53], which agrees with our results, although they fell short of significance (*p* < 0.10; Fig 2d). Studies such as Pederson et al. [54] indicate the importance of winter temperatures to North American conifers. In general, our study does not agree with other works showing a positive association between MXD chronologies and summer temperatures in parts of the Northern Hemisphere [e.g. 39,42,50]. However, this could be attributable to *P*. *cooperi* sensitivity to high summer temperatures, which adversely affects their radial growth and may lead to physiological impairment [24], affecting the cell formation of latewood.

### MXD Chronologies show a link to ENSO

MEI teleconnections with radial growth of *P*. *cooperi* show principal association with the previous winter and early spring. This trend may be caused by ENSO phase change [55]. Radial growth likely improves with positive values of ENSO, affecting precipitation. This results in higher atmospheric water vapor and moisture available to soil for cambial reactivation [56]. These teleconnections have been demonstrated in northern Mexico by Seager et al. [48] and are consistent with the present results. Patterns of spatial correlation between regional chronology of the MXD and MEI variables (SST, SLP, AST, U250 and V250) in December–February show how the climate variability of ENSO influences the growth of *P*. *cooperi* in northern Mexico, mainly near the central tropical Pacific Ocean (Fig 3). Sea surface temperature and SAT show classic ENSO patterns with an increase of these variables (warm anomalies), and increasing rainfall during those months, which positively affects species growth. Wind patterns at the 250 hPa level indicate decreased flow from west to east (westerlies) of U250, specifically in the central tropical Pacific, and increased flow from north to south of V250, specifically in the central and eastern tropical Pacific during El Niño events. Such changes affect the growth of trees in the Sierra Madre Occidental. Regarding the association of SLP with radial growth of *P*. *cooperi*, it appears to be characterized by a dipole between the eastern and western tropical Pacific more than by the Niño3.4 region, with both negative and positive values.

### Carbon uptake by tree rings

Annual wood density is directly related to increased rates of carbon assimilation (photosynthetic rate), and inversely related to stomatal conductance [57]. The latter depends on high values of air temperature and, particularly, on the amount of water available and transpiration rates. Therefore, at high elevation (HE) sites, the positive relationship of MXD with winter minimum temperature and precipitation suggests a relationship between stomatal conductance during the period in which photosynthesis is producing latewood. During photosynthesis, trees attempt to optimize the relationship between water loss and carbon capture, which is controlled primarily by the stomatal function according to the difference in vapor pressure [58]. Water deficit at high elevation sites appears to strongly affect radial growth and, thus, carbon sequestration. This can be interpreted as an important reserve of water, which does not necessarily mean water sufficiency but rather positive water balance [59]. In physiological and anatomical terms, this is explained by thicker tracheids.

Analyzing the spatial variation in the aboveground biomass, we observed that the higher site (HE) was that obtained a greater carbon accumulation (Fig 5, Table 3), which was influenced by high values of basal area and wood density (Fig 4a and 4b). There was a strong relation among these variables (Fig 6). Variation in annual density is mainly related to biomass increment and physiological processes related to carbon assimilation, allocation and use [4,39]. This higher wood density of site HE (respect to site H), can be explained by an ecological response of trees to adverse weather conditions, which would cause a change in the cellular structure affecting the within-ring wood density profile [60]. In this context, climatic forcing of wood density is not necessarily restricted to the late growing season only, since that strong associations may exist during a major part of the growing season [61].

Strong sensitivity to precipitation of *P*. *cooperi* at HE can be attributed to increased formation of cell walls [59]. *P*. *cooperi* at that site appears to be resistant to drought, which leads to an increase in the formation of latewood and, therefore, wood density. That is, drought stress can reduce carbohydrate assimilation during the season prior to growth [62]. High evaporation rates can induce breathing and evapotranspiration of the stand, causing water deficits [63]. Thus, we suggest that the positive relationship between wood density and minimum temperature of the previous winter is a consequence of the negative effect on radial tree growth. These delayed responses underline the importance of climatic conditions controlling evaporative demand prior to the start of that growth [53]. We have shown that MXD is very sensitive to water deficit and high evaporation rates, suggesting that this evapotranspiration demand is a major factor in carbon capture. Recent findings suggest that increasing atmospheric water vapor demand is a crucial factor in forest decline [64].

These findings regarding wood formation refine our knowledge of the magnitude and dynamics of carbon sequestered in *P*. *cooperi*. This has direct implications for the productivity of ecosystems in which *P*. *cooperi* grows, and greatly reduces the uncertainty in modeling terrestrial carbon. We found that the cold season—prior to growth—largely controls carbon sequestration, particularly at higher altitude site (with greater productivity) [40]. Fig 4 shows greater temporal carbon variability at site HE compared with site H, and therefore increased vulnerability to extreme climatic variations. Monitoring winter conditions prior to growth is therefore vital, and will become more so for future warm events. Evidence is mounting that climate extremes such as droughts can lead to a decrease in regional ecosystem carbon stocks and therefore have the potential to negate an expected increase in terrestrial carbon uptake [40].

### Implications of the study

The function of biomass used is appropriate for the study area [15]. However, combining densitometry with allometry (biometric techniques) has substantially improved the accuracy of carbon calculation. Assuming a uniform density according to tree age is incorrect [65]. In a recent paper, Taki et al [66] found differences of 6% to 27% in carbon estimation using conventional methods. They concluded that these differences directly affect global and regional estimates of carbon sinks, including management practices [67]. Estimation errors derived from the composition and distribution of species at the time of sampling are typically discarded [1]. Sometimes dendrochronological sampling may skip aspects such as the density and structure of the mass, which can bias conclusions regarding forest productivity [68].

The carbon dynamics, based on the temporal variability of wood density, represents a significant improvement over previous studies on carbon sequestration [69]. In particular, understanding density variation as a function of radial growth will facilitate greater knowledge about how to accurately estimate carbon sequestration, which could be useful for environmental mitigation strategies. Therefore, *P*. *cooperi* is a more important ecological species than has been recognized heretofore. Wood density variations affect not only tree growth but also wood properties, particularly in species with great density differences between early and latewood [13]. These physiological processes are relevant for *P*. *cooperi*, because it is an industrial species widely used in northern Mexico for its mechanical properties [70]. There is an urgent need to further investigate species that have traditionally been used to estimate biomass and carbon. This is a complex task that involves using tree rings as a key element to study forest biomass dynamics, representing an opportunity to better understand the mechanisms of uptake mass carbon. Therefore, the inclusion of temporal variability of wood density improves substantially knowledge to estimate carbon sequestration over time, especially in regions where there are no permanent plots, and also, we demonstrate that maximum density latewood of *P*. *cooperi* has a high sensitivity to local and large-scale climate variability, contributing to future climate change studies in Northern Mexico.
